# Constructing the Mo_2_C@MoO_x_ Heterostructure for Improved SERS Application

**DOI:** 10.3390/bios12020050

**Published:** 2022-01-19

**Authors:** Kui Lai, Kaibo Yuan, Qinli Ye, Anqi Chen, Dong Chen, Da Chen, Chenjie Gu

**Affiliations:** 1 The Research Institute of Advanced Technologies, Ningbo University, No. 818 Fenghua Road, Ningbo 315211, China; laikui18839135662@163.com (K.L.); guchenjie@nbu.edu.cn (C.G.); 2 School of Physical Science and Technology, Ningbo University, No. 818 Fenghua Road, Ningbo 315211, China; 15836294399@163.com (K.Y.); yql1511362567@126.com (Q.Y.); chenda@nbu.edu.cn (D.C.)

**Keywords:** SERS, molybdenum carbide, molybdenum oxide, heterojunctions, charge transfer

## Abstract

Surface-enhanced Raman scattering (SERS) is a non-destructive spectra analysis technique. It has the virtues of high detectivity and sensitivity, and has been extensively studied for low-trace molecule detection. Presently, a non-noble-metal-based SERS substrate with excellent enhancement capabilities and environmental stability is available for performing advanced biomolecule detection. Herein, a type of molybdenum carbide/molybdenum oxide (Mo_2_C@MoO_x_) heterostructure is constructed, and attractive SERS performance is achieved through the promotion of the charge transfer. Experimentally, Mo_2_C was first prepared by calcinating the ammonium molybdate tetrahydrate and gelatin mixture in an argon atmosphere. Then, the obtained Mo_2_C was further annealed in the air to obtain the Mo_2_C@MoO_x_ heterostructure. The SERS performance was evaluated by using a 532 nm laser as an excitation source and a rhodamine 6G (R6G) molecule as the Raman reporter. This process demonstrates that attractive SERS performance with a Raman enhancement factor (EF) of 1.445 × 10^8^ (R6G@10^−8^ M) and a limit of detection of 10^−8^ M can be achieved. Furthermore, the mechanism of SERS performance improvement with the Mo_2_C@MoO_x_ is also investigated. HRTEM detection and XPS spectra reveal that part of the Mo_2_C is oxidized into MoO_x_ during the air-annealing process, and generates metal–semiconductor mixing energy bands in the heterojunction. Under the Raman laser irradiation, considerable hole–electron pairs are generated in the heterojunction, and then the hot electrons move towards MoO_x_ and subsequently transfer to the molecules, which ultimately boosts the Raman signal intensity.

## 1. Introduction

Surface-enhanced Raman spectroscopy (SERS) is a single-molecule analytical technique that can detect and identify chemical and biological compounds through their unique Raman vibration fingerprints [1]. Presently, it has been widely implemented in the fields of bio-medicine, homeland security, food safety, and medical diagnosis, etc. [2,3,4,5,6]. Although the exact mechanism of the enhancement effect is still under debate, the presence of an electromagnetic mechanism (EM) and a chemical mechanism (CM) are two commonly accepted enhancement theories [7]. Generally, the EM relies on the generation of surface plasmon resonance (SPR) on the noble metal-based SERS substrates, which subsequently amplifies the oscillating dipoles of the molecules and ultimately produces the enhanced Raman signal intensity. On the other hand, the CM relies on the photo-induced charge transfer between the molecules and the SERS substrate. The extra charges amplify the polarizability of the molecules, and consequently enhance the Raman signal intensity. Typically, the enhancement factor (EF) produced by the EM can reach 10^6^ or even higher, whereas it is only around 10–1000 times that obtained on the conventional CM-dependent SERS substrates [8,9].

Up to today, an enormous number of studies on noble metal-based (Au, Ag, and Cu) SERS substrates have been performed. By designing and preparing noble metal-based nanostructures with various morphologies or compositions, fantastic SERS performance has been demonstrated [10,11,12]. At present, it is well accepted that precise control of the noble metal morphologies and compositions is a major necessity for generating strong plasmonic coupling; however, manufacturing the large-scale nanostructures with high accuracy requires excessive cost. In addition, noble metals generally exhibit poor stability and biocompatibility; thus, SERS detectors prepared with noble metals are rarely used in biological applications [9].

Recently, a non-noble-metal-based SERS substrate has attracted an enormous amount of attention [13,14,15,16]. Significantly, molybdenum-based materials, such as molybdenum disulfide (MoS_2_), molybdenum telluride (MoTe_2_), and molybdenum sub-oxide (MoO_x_), show promising SERS performance, which results from the exceptional charge transfer capability caused by the defects in the material [17,18,19]. For example, a kind of two-dimensional molybdenum disulfide (MoS_2_) was used to construct the nonmetallic SERS-based immunoassay. In addition, a desirable EF of 10^5^ was obtained by the efficient charge transfer resonances induced by the 532 nm laser excitation, which has been ascribed to the effective enrichment of molecules on the large active surfaces of MoS_2_ [20]. Meanwhile, a metallic MoO_2_ was prepared via the hydrothermal method, and the evaluation of its SERS performance revealed that an EF of 3.75 × 10^6^ can be achieved due to the excellent charge transfer capability of the MoO_2_ [21]. On the other hand, recent studies have also introduced heterojunctions to promote the charge transfer on the non-noble-metal-based SERS substrate, and they have shown that heterojunction tends to help to efficiently transfer the photo-generated free carriers to the molecules, which significantly improves the SERS performance. Specifically, defective molybdenum oxide/tungsten oxide (MoO_x_/WO_x_) are prepared, then they are mixed with different weight ratios to construct the nano-heterojunctions. This shows that an attractive EF of 10^8^ can be obtained on the substrate with optimal mixing ratios [22]. However, tungsten suboxide suffers from issues of environment instability when the above method is used in a harsh condition or when the material is kept in the air for a long time [23]. At this point, it is notable that improving the charge transfer between the molecules and substrates is a key factor for promoting the SERS performance of the non-noble-metal-based substrate. Moreover, the environmental stability of the SERS substrate material is also essential for various detection applications.

Herein, a molybdenum carbide/molybdenum oxide (Mo_2_C@MoO_x_) heterostructure is constructed to promote its charge transfer for SERS applications. Experimentally, Mo_2_C was first prepared by calcinating the ammonium molybdate tetrahydrate and gelatin mixture in an argon atmosphere. Then, the obtained Mo_2_C was oxidized in the air to obtain the Mo_2_C@MoO_x_ heterostructure. The SERS performance was evaluated by using a 532 nm laser as the excitation source and a rhodamine 6G (R6G) molecule as the Raman reporter. Attractive SERS performance with a Raman enhancement factor (EF) of 1.445 × 10^8^ (R6G@10^−8^ M) and a limit of detection of 10^−8^ M was achieved. Furthermore, the SERS performance improvement of the Mo_2_C@MoO_x_ is also investigated. High-resolution TEM detection and XPS spectra reveal that part of the Mo_2_C is oxidized into MoO_x_ during the air-annealing process, and generates high levels of metal–semiconductor mixing energy in this hetero-region. Under Raman laser irradiation, the enhanced light absorption produces substantial electron–hole pairs in the hetero-region, and then these electrons move towards the molecules because of the MoO_x_ energy level, which ultimately boosts the Raman signal intensity.

## 2. Materials and Methods

### 2.1. Chemicals

Commercial Mo_2_C (CAS No.: 12069-89-5, 99.95%) was purchased from Maclin Biochemical Co., Ltd., Shanghai, China. Gelatin (CAS No.: 9000-70-8, 99%) was obtained from Macklin Biochemical Co., Ltd., Shanghai, China. Ammonium molybdate tetrahydrate (CAS No.: 12054-85-2, AHM, >99%) was purchased from Sinopharm Chemical Reagent Co., Ltd., Shanghai, China. All chemicals were used without further purification. Deionized water (resistivity of 18.2 MΩ•cm) was used to prepare the solutions in all the experiments.

### 2.2. Synthesis of Mo_2_C

To obtain the Mo_2_C, in the experiment, gelatin (1 g) was first added into deionized water (20 mL), and then AHM (2 g) was put into the solution. After that, the above mixture was stirred vigorously in a 50 °C water bath for 2 h. Thereafter, the prepared solution was dried in the oven to drive the excessive water out at 80 °C for another 25 h. Next, the mixture was dehydrated at 200 °C in the quartz tube with argon flushing for 1 h. Thereafter, the temperature of the quartz tube was raised to 900 °C by 10 °C/min, and held at that temperature for another 1 h. Finally, the quartz tube was cooled down naturally. In the entire carbonization process, the argon gas flow was kept at 45 mL/min. The obtained black solid products were washed and centrifuged with distilled water several times to remove the residue of the reactants, and finally dried in vacuum at 80 °C for further use.

### 2.3. Preparing the Mo_2_C@MoO_x_ Films

The obtained Mo_2_C powder was dispersed in alcohol, and ultrasonicated for 15 min. Then, 10 μL of the mixed solution was dropped onto the silicon wafer that was pretreated with the piranha solution and dried in the air. Finally, the silicon wafer was transferred to the hot plate for annealing. The optical image and cross-section SEM image of the prepared substrate are shown in Figure S1. It can be observed that the physical thickness of the Mo_2_C is about 78.7 nm.

### 2.4. Measurements and Characterization

X-ray diffraction (XRD) spectra were recorded on a D8 Advance diffractometer equipped with a Lynxeye Xe detector (Bruker AXS, Karlsruhe, Germany). X-ray photoelectron spectroscopy (XPS) was measured with a thermal K-α instrument, the X-ray emission source is Al-K-α rays (hν ≈ 1486.6 eV), and the experimental binding energy data are corrected to C 1 s = 284.8 eV. The scanning electron microscope (SEM) images of the prepared material were detected on an SU-70 field emission scanning electron microscope (SU-70, Hitachi, Japan) under an accelerating voltage of 5 kV. For the transmission electron microscopy (TEM), high-resolution TEM images were obtained using the transmission electron microscope (Tecnai G2F20S-Twin, FEI, Hillsboro, OR, USA) under an accelerating voltage of 200 kV. UV–vis spectra were recorded on the spectrometer (TU1901, P-General, Samutprakarn, Thailand). The SERS performance was examined using a ProSp-Micro40-VIS Raman system (Hangzhou SPL, Hangzhou, China), and QE Pro spectrometer (QE pro, Ocean Optics, Dunedin, FL, USA) was used to record the Raman spectra. The excitation laser wavelength was 532 nm and the power on the substrate was 1 mW. Meanwhile, the integration time was set to 10 s, and the objective lens was 50×.

## 3. Results and Discussion

**Figure 1:**
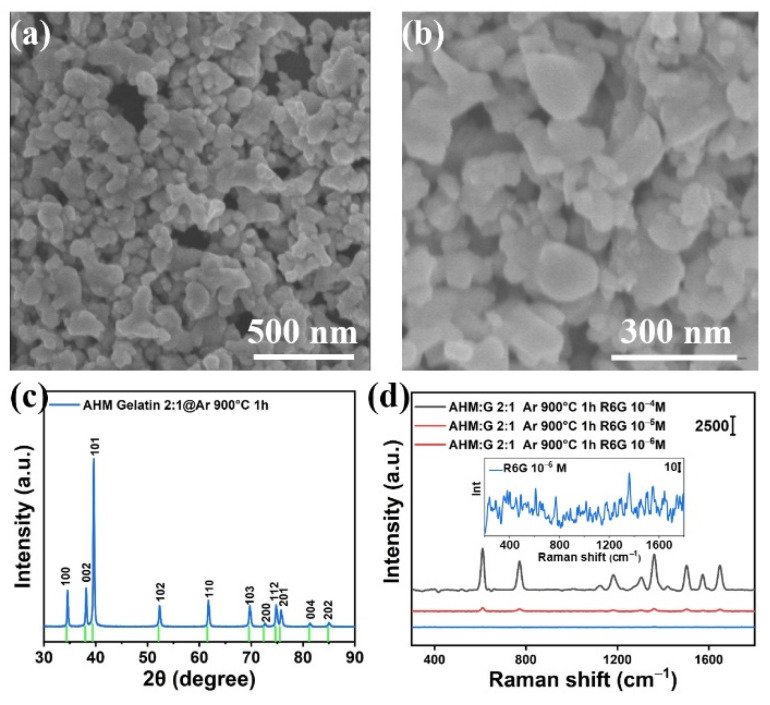
(**a**,**b**) The SEM images of the synthesized Mo_2_C with different magnifications; (**c**) the XRD spectrum of the synthesized Mo_2_C; (**d**) the SERS spectra of R6G measured on the synthesized Mo_2_C.

The black Mo_2_C powder was first detected via SEM. Figure 1a,b shows the microscopic morphologies of the Mo_2_C at different magnifications. It can be seen that the Mo_2_C nanoflakes are in irregular shapes, and the surface of the nanoflakes is relatively smooth. The size of a typical nanoflake is about 87 × 120 nm^2^. The crystal structure of Mo_2_C was then identified via XRD. Figure 1c shows that sharp diffraction peaks can be determined, which indicates that the prepared Mo_2_C has good crystallinity. Then, the SERS performance of the synthesized Mo_2_C was evaluated by using the R6G as the Raman reporter. As Figure 1d shows, the characteristic Raman vibrational peaks can be observed when a 10^−4^ M R6G solution is used. However, when the R6G concentration is reduced to 10^−5^ M, the Raman peak intensities reduce significantly, and the Raman peaks even become completely extinct when the concentration of R6G solution reaches 10^−6^ M (see inset of Figure 1d). Based on the above observation, it is evident that the SERS performance of the synthesized Mo_2_C is extremely weak, which could be ascribed to the weak charge transfer capability from the substrate to the molecules.

**Figure 2:**
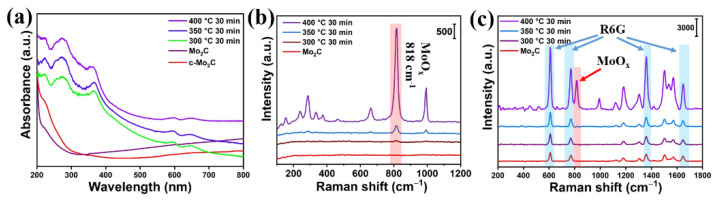
(**a**) The absorption curves measured on different materials that were annealed under different temperatures; (**b**) the corresponding Raman spectrum of the materials annealed at different temperatures; (**c**) the SERS spectrum of R6G measured on different materials that were annealed at different temperatures.

In order to improve the SERS performance of the synthesized Mo_2_C, the Mo_2_C@MoO_x_ heterostructure was constructed by annealing the Mo_2_C powder in the air. Firstly, three sets of Mo_2_C powder were subjected to air annealing at 300 °C, 350 °C, and 400 °C, with the time fixed at 30 min. The UV–vis absorption spectra of the products were collected, and these are shown in Figure 2a. The figure shows that the synthesized Mo_2_C exhibits metallic properties [24]. Meanwhile, the absorption curve tails up when compared to that measured on the commercial Mo_2_C in the long wavelength range, which is ascribed to the existing of defects in the synthesized Mo_2_C [25]. On the other hand, the absorption edges of the annealed products were significantly shifted to longer wavelengths, which suggests that the band gap opened up due to the formation of MoO_x_ [26]. Additionally, the Raman spectra of the products were also measured. As can be observed in Figure 2b, only weak intrinsic Raman peaks of MoO_x_ can be observed on the spectra collected for the materials annealed at 300 °C and 350 °C, revealing that the thermostability of Mo_2_C is quite good. On the other hand, prominent Raman peaks appear on the material annealed at 400 °C. Specifically, the Raman peak which appears at 288 cm^−1^ is assigned to the wagging mode of the double bond O=Mo=O, while the Raman peaks that appear at 662, 818, and 994.7 cm^−1^ are assigned to the stretching mode of the triply coordinated oxygen (3Mo-O), the doubly coordinated oxygen (2Mo-O), and the terminal oxygen (Mo^6+^-O), respectively. Finally, the Raman peaks that show at 243, 286, 335, and 375 cm^−1^ are indexed to the oscillation modes of (2Mo-O), (O=Mo=O), (3Mo-O), and (Mo=O), respectively [22]. The above observation demonstrates that the oxidization reaction at 400 °C is more efficient at oxidizing the Mo_2_C into MoO_X_. Furthermore, the SERS performance of the materials oxidized at different temperatures was investigated, and 10^−4^ M R6G was used as the probe molecule. Figure 2c shows that the characteristic peaks of the R6G, from 600 to 2000 cm^−1^, could be observed on all films. In detail, the Raman peaks that appear at 608 and 772 cm^−1^ are attributed to aromatic bending, and the Raman peak that is located at 1183 cm^−1^ arises from aromatic C-H bending. Meanwhile, the Raman peak that shows at 1362 cm^−1^ is assigned to C-C bridge stretching and, finally, the Raman peaks that appear at 1507 and 1646 cm^−1^ are ascribed to aromatic C-C stretching [27]. More importantly, it also can be observed that the SERS intensities of R6G measured on the Mo_2_C annealed at 300 °C and 350 °C are similar to that measured on the synthesized Mo_2_C, which suggests that there is almost no change in the charge transfer capability, even when the Mo_2_C has been annealed in the air. However, it is interesting to observe that the SERS intensities of R6G measured on the Mo_2_C annealed at 400 °C are almost two times high than the intensities measured on the other two mentioned above, which reveals that the charge transfer capability of the Mo_2_C annealed at 400 °C is significantly improved.

**Figure 3:**
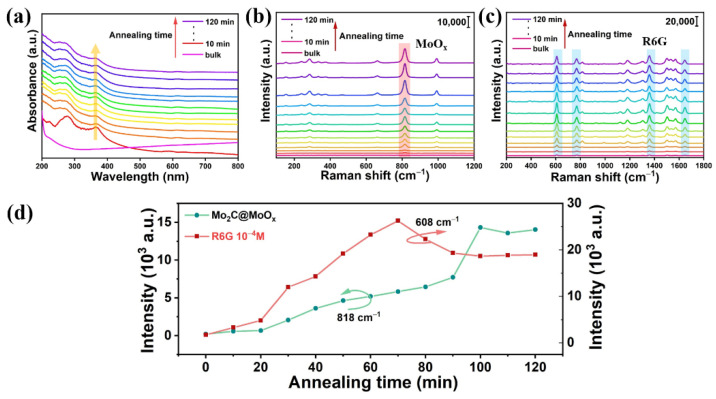
(**a**) The absorption curves measured; (**b**) the corresponding Raman spectrum; (**c**) the SERS spectrum of R6G measured on different samples that were annealed for different lengths of time; (**d**) the evolution of the Raman peak (818 and 608 cm^−1^) intensities with different annealing times.

To further explore the effect of the annealing time on Mo_2_C’s SERS performance, the synthesized Mo_2_C powder was subjected to different annealing times, while the temperature was fixed at 400 °C. As Figure 3a shows, the absorption peak blue shifts with the extension of the annealing time, which suggests that there is an increase in the amount of the MoO_x_ component. Meanwhile, the Raman spectra of the annealed materials and the corresponding SERS spectra of the R6G were also recorded, and these are shown in Figure 3b,c, respectively. The evolutions of the Raman peak intensities at 818 cm^−1^ (MoO_x_) and 608 cm^−1^ (R6G) are extracted and shown in Figure 3d. This illustrates that the characteristic Raman peaks of MoO_x_ gradually increase with the extension of the annealing time, which further confirms that the percentage of MoO_x_ increases in the products. However, the peak intensity saturates when the annealing time is >100 min, indicating that the Mo_2_C has been fully oxidized into MoO_3_. At the same time, for the Raman peak of R6G, the Raman peak intensity gradually climbs up in the beginning, and it reaches the maximum point when the annealing time is set to 70 min. However, further increasing the annealing time degrades the material’s SERS performance, and the SERS enhancement capability remains unchanged when the annealing time is >100 min, which verifies that the Mo_2_C is fully oxidized at that time.

**Figure 4:**
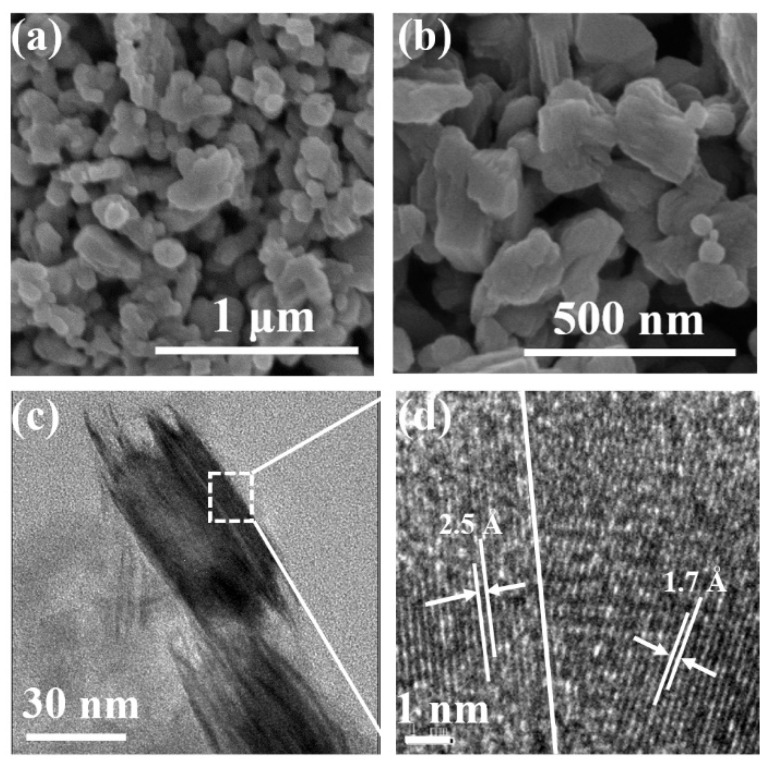
(**a**,**b**) Different magnifications of the SEM images of the Mo_2_C annealed at 400 °C for 70 min; (**c**) the TEM image of the Mo_2_C annealed at 400 °C for 70 min; (**d**) the corresponding high-resolution TEM image of the Mo_2_C annealed at 400 °C for 70 min.

**Figure 5:**
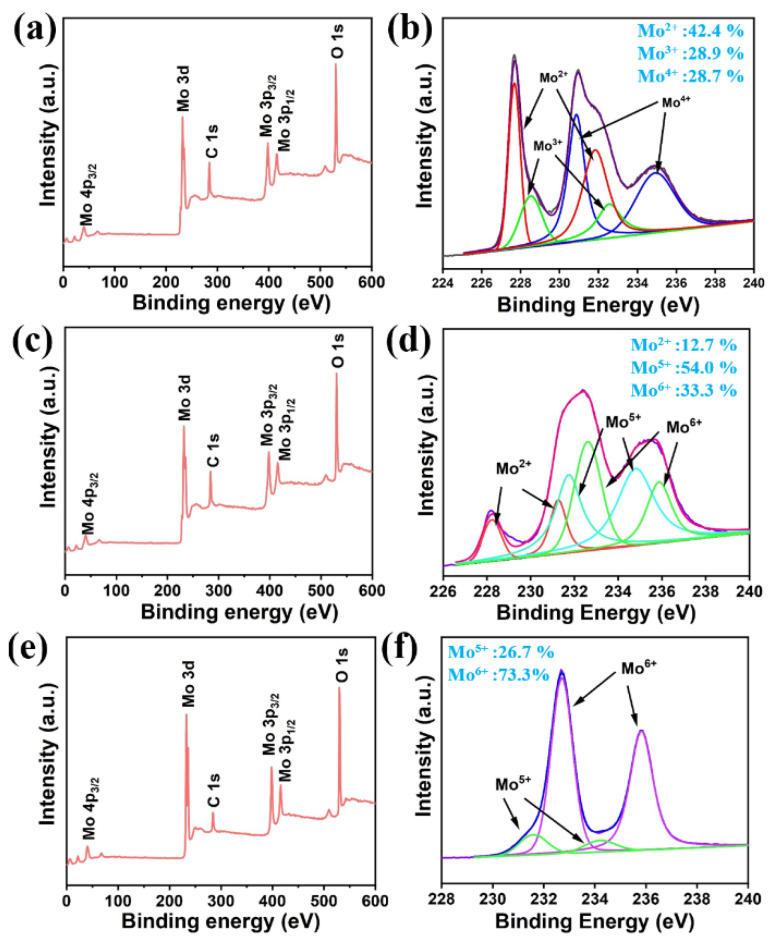
(**a**) The XPS element scanning spectrum measured on the synthesized Mo_2_C; (**b**) the high-resolution XPS scanning of the Mo 3d orbit on the synthesized Mo_2_C; (**c**) the XPS element scanning spectrum measured on the Mo_2_C@MoO_x_; (**d**) the high-resolution XPS scanning of the Mo 3d orbit on the Mo_2_C@MoO_x_; (**e**) the XPS element scanning spectrum measured on the MoO_3_; (**f**) the high-resolution XPS scanning of Mo 3d orbit on the MoO_3_.

Next, the discussion will focus on the mechanism of the SERS performance improvement observed for the Mo_2_C annealed at 400 °C for 70 min. Firstly, SEM and TEM was used to detect the morphologies of the material. It can be seen on the SEM images in Figure 4a,b that the product still retains the irregular shapes as that of Mo_2_C, but the surface becomes rough, which could be ascribed to the formation of the MoO_x_. Meanwhile, the TEM image in Figure 4c,d reveals more details. It shows that the edge of the particle forms tiny antennas. In addition, the high-resolution transmission electron microscope (HRTEM) image in Figure 5d displays two lattice fringes. The first one shows d-spacing of 2.5 Å, which is consistent with the (002) plane of Mo_2_C. The second one shows lattice fringes of 1.7 Å, which corresponds to the (211) plane of MoO_3_. This physical evidence indicates that annealing in the air forms the Mo_2_C@MoO_3_ heterostructure.

In addition, X-ray photoelectron spectroscopy (XPS) was used to analyze the valence states of the elements. Figure 5 shows the XPS spectra collected for the synthesized Mo_2_C, Mo_2_C@MoO_x_ (400 °C for 70 min), and MoO_x_ (400 °C for 100 min), respectively. Clearly, the respective peaks belonging to the Mo, O, and C atoms are observed on the element scanning spectra. Moreover, Figure 5b shows the high-resolution spectra of the Mo 3d that was measured on the synthesized Mo_2_C. Three peak-shaped spectrum can be observed. Then, deconvolution analysis was performed. The two spin-orbit doublets at 227.63 and 228.33 eV can be assigned to the Mo^2+^ ion, the double peaks at 230.78 and 232.18 eV correspond to the Mo^4+^ ions, while the doublets appearing at 231.43 and 234.83 eV are indexed to the Mo^5+^ ions. Moreover, it can be attained that 42.4% of the Mo atoms are in a Mo^2+^ state, indicating that they form the Mo_2_C structure. On the other hand, it also shows that the rest of the Mo ions are still in a high valence state (Mo^3+^:28.9%, Mo^4+^:28.7%), which reveals the possible existence of impurities (e.g., MoC) in the product [28]. Furthermore, the high-resolution XPS spectra of the Mo_2_C@MoO_x_ is shown in Figure 5d. This analysis shows that the percentage of Mo^2+^ component is reduced to 12.7% after the annealing process. Correspondingly, the percentage of Mo high valence states increases. Specifically, the percentage of Mo^5+^ ions increases to 54.0%, while Mo^6+^ ions also appear, and they make up over 33.3% of the total Mo atoms. The above change suggests that Mo_2_C is partially oxidized and forms an optimal heterostructure with excellent charge transfer capabilities. The high-resolution spectra of the MoO_x_ are shown in Figure 5f. Clearly, a two-peak-shaped spectrum is obtained. Through the analysis, it is found that the sample is mainly composed of Mo^6+^ ions (73.2%), while only a small percentage of the atoms are in a Mo^5+^ state (26.7%), which further confirms that the Mo_2_C is completely converted to MoO_3_ [29]. At this time, the mechanism of the SERS enhancement observed on Mo_2_C@MoO_x_ can be understood in Figure S2. It can be seen that the metal–semiconductor generates high levels of mixing energy in the hetero-region, the incident light produces substantial numbers of electron–hole pairs, and then the hot electrons are separated (Figure S3) and move towards the molecules because of the MoO_x_ energy levels, which ultimately boosts the Raman signal intensity [25,30,31].

**Figure 6:**
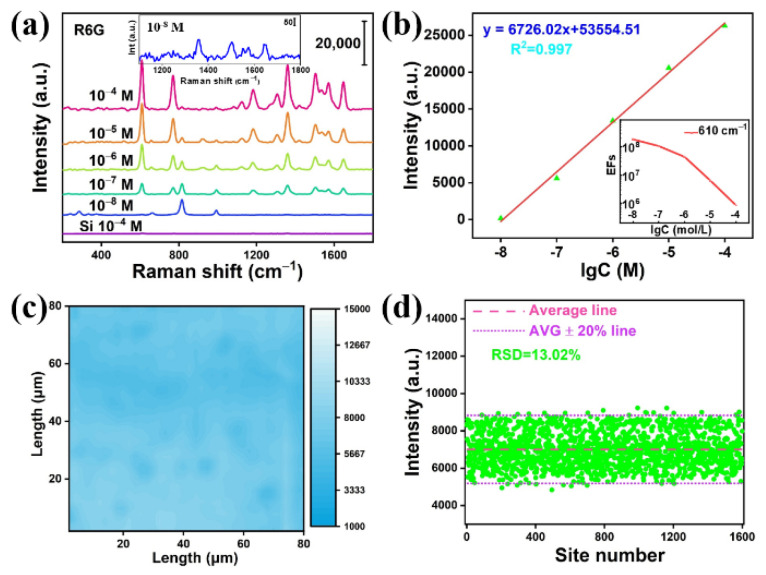
(**a**) The SERS spectrum of the R6G measured with different concentrations; (**b**) the logarithmic plot of the Raman peak (608 cm^−1^) intensity versus different R6G concentrations; (**c**) the Raman peak (608 cm^−1^) intensity mapping; (**d**) the statistical data of the mapped data points.

Finally, the sensitivity of the Mo_2_C@MoO_x_ heterostructure was also evaluated. Figure 6a shows the collected Raman spectra when the R6G concentrations decrease from 10^−4^ to 10^−5^, 10^−6^, 10^−7^ and to 10^−8^ M, respectively. Figure 6a shows that the Raman signal of the R6G is still detectable when the concentration reaches 10^−8^ M, which indicates that the detection limit of the Mo_2_C@MoO_x_ heterostructure can be as low as 10^−8^ M. In addition, Figure 6b shows the logarithmic plot of the Raman peak intensity (608 cm^−1^) versus the R6G concentration from 10^−4^ to 10^−8^ M. Clearly, it can be seen that the substrate has a good linear detection capability. Other than that, the enhancement factor (EF) of the Mo_2_C@MoO_x_ film at different R6G concentrations is also calculated by integrating the peak intensity at 608 cm^−1^ (see the Supplementary Materials). It can be seen from Figure 6b that EFs of 8.4 × 10^5^ (10^−4^ M R6G), 6.4 × 10^6^ (10^−5^ M R6G), 4.4 × 10^7^ (10^−6^ M R6G), 1.0 × 10^8^ (10^−7^ M R6G), and 1.8 × 10^8^ (10^−8^ M R6G) can be obtained. At this moment, it is worth noting that the EF increases with the decrease in the R6G concentration, which could be ascribed to the improvement of the charge efficiency when less molecules are adsorbed on the surface. Other than that, other Raman reporters, such as methylene blue (MB), were also used. It can be seen from Figure S4 that the substrate shows excellent Raman enhancement capabilities. Moreover, the LoD can reach 10^−8^ M, as well. Furthermore, the uniformity of the Raman signal intensity on the Mo_2_C@MoO_x_ heterostructure was evaluated. To achieve this, the Raman signal intensity map was recorded for the Mo_2_C@MoO_x_ substrate. Specifically, a randomly selected square of 80 × 80 μm^2^ was used for the mapping measurement. In the measurement, 10^−4^ M R6G was used as the reporter and a total of 1600 points were collected. Figure 6c shows the intensity mapping of the Raman peak at 608 cm^−1^. The figure shows good Raman signal intensity for a large-scale area. In addition, the statistical data in Figure 6d show that the average mapping intensity is 7004.5 cps, while the relative standard derivative (RSD) is calculated to be 13.02%. The above data indicate that the prepared Mo_2_C@MoO_x_ substrate has excellent uniformity. Finally, as the literature reported that both Mo_2_C and MoO_x_ have relatively good resistance to high temperatures and acidic/alkaline environments, it is expected that the Mo_2_C@MoO_x_ heterostructure-based SERS substrate can expand the potential application fields for multipurpose detection [21,25].

## 4. Conclusions

To summarize, a type of Mo_2_C@MoO_x_ heterostructure has been finely prepared, and improved SERS performance has been achieved through the heterostructure-induced charge transfer. Specifically, attractive SERS performance with a Raman enhancement factor (EF) of 1.445 × 10^8^ (R6G@10^−8^ M) and a limit of detection of 10^−8^ M has been achieved. Furthermore, the mechanism of the SERS performance improvement was also investigated. HRTEM detection and XPS spectra revealed that part of the Mo_2_C is partially oxidized into MoO_x_, and generates metal–semiconductor mixing energy bands in the heterojunction. Under Raman laser irradiation, a considerable number of hole–electron pairs are generated in the heterojunction, and then the hot electrons move towards MoO_x_ and subsequently transfer to the molecules, which ultimately boosts the Raman signal intensity.

## Data Availability

The data presented in this study are available in article or supplementary material.

## Supplementary Materials

The following supporting information can be downloaded at: https://www.mdpi.com/article/10.3390/bios12020050/s1, Figure S1: The optical and cross-section SEM image of the deposited Mo_2_C film; Figure S2: The charge transfer path; Figure S3: PL spectra measured on Mo_2_C and Mo_2_C@MoO_x_; Figure S4: SERS spectra of MB; Note 1: calculation of the enhancement factor.
