# Supplementary material for: Constructing the Mo2C@MoOx Heterostructure for Improved SERS Application
Source: Biosensors (Basel). 2022 Jan 19;12(2):50. doi: 10.3390/bios12020050 (PMC8869368; doi:10.3390/bios12020050)
Supplement: Supplementary file 1 [file biosensors-12-00050-s001.zip › biosensors-1547646-supplementary.pdf]

biosensors

MDPI

Supplementary Materials: **Constructing the $Mo_2C@MoO_x$ Heterostructure for Improved SERS Application**

Kui Lai [1,2], Kaibo Yuan [2], Qinli Ye [2], Anqi Chen [1,*], Dong Chen [2,*], Da Chen [2] and Chenjie Gu [1,2]

[1] The Research Institute of Advanced Technologies, Ningbo University, No. 818 Fenghua Road, Ningbo 315211, China; laikui18839135662@163.com (K.L.); guchenjie@nbu.edu.cn (C.G.)
[2] School of Physical Science and Technology, Ningbo University, No. 818 Fenghua Road, Ningbo 315211, China; 15836294399@163.com (K.Y.); yql1511362567@126.com (Q.Y.); chenda@nbu.edu.cn (Da.C.)
* Correspondence: chenanqi@nbu.edu.cn (A.C.); chendong@nbu.edu.cn (Do.C.)

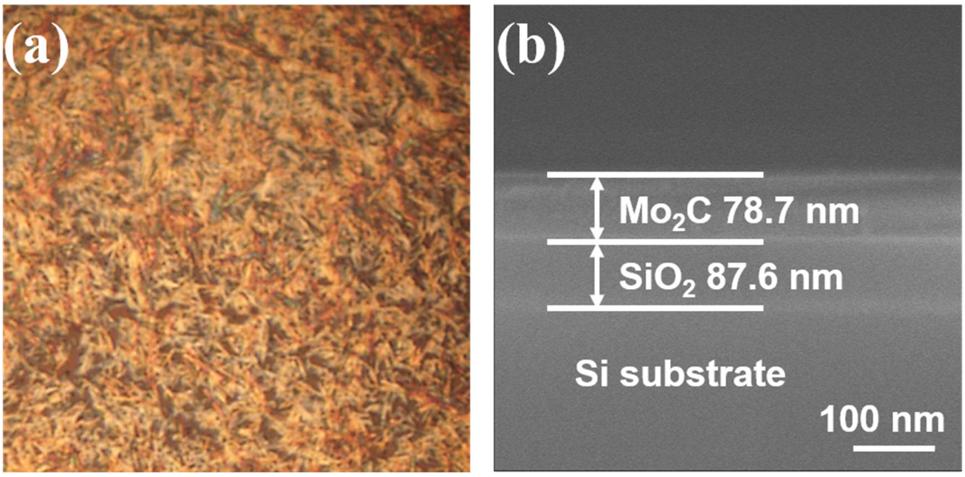

Figure S1. (**a**) The optical image; (**b**) the cross-section SEM image the deposited Mo_2C film on Si substrate.

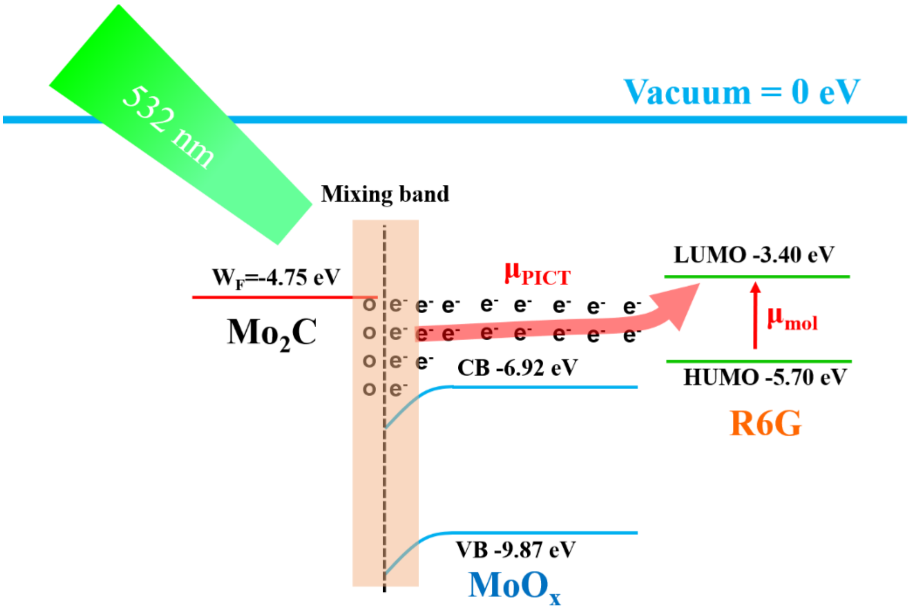

Figure S2. The proposed charge transfer path on the Mo_2C/MoO_x heterostructure.

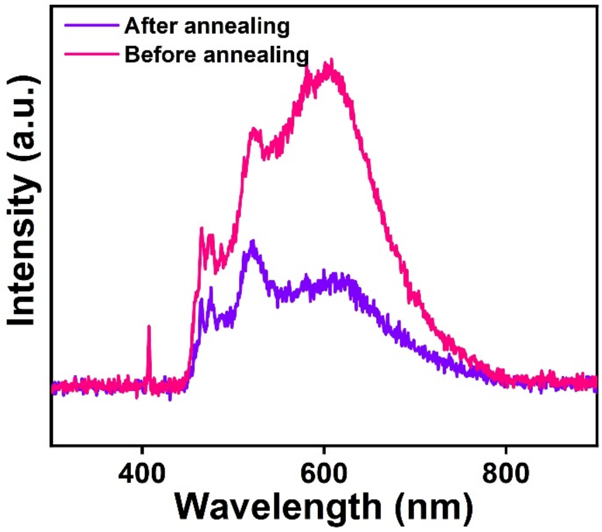

Figure S3. The photoluminance (PL) spcetra measured on Mo_2C and $Mo_2C@MoO_x$. The reduction of PL intensity indicates the separation of hole-electorn pair.

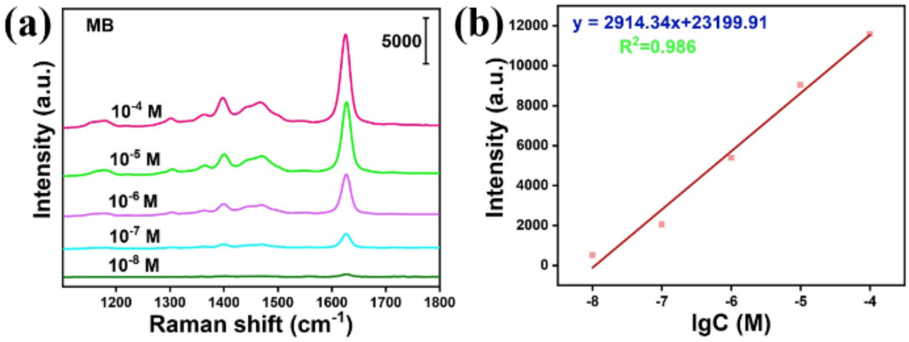

Figure S4. (**a**) the SERS spectra of MB measured on Mo_2C/MoO_x; (**b**) the logarithmic plot of Raman peak (1626 cm^{-1}) intensity versus different MB concentrations.

Calculation of the Enhancement Factor:

The enhancement factor is calculated by the following equation:

$$EF = (I_{SERS}/I_{bulk})\times(N_{bulk}/N_{SERS}) \quad (1)$$

where I_{SERS} and I_{bulk} represent the integrated intensities of SERS peaks at 608 cm^{-1} of R6G molecules on the film and R6G powder, respectively. N_{bulk} and N_{SERS} are the number of R6G in the powder and on the films within the laser spot, respectively. To calculate the N_{bulk}, in the first step, 10 ul of 50 mM R6G ethanol solution is dropped on the surface of the Si chip with the size of 5 × 5 mm^2 (S), drying in the air. Then, according to the measurement settings (50× objective lens, 532 nm excitation source, 1 mW laser power and 10 s integration time), the N_{bulk} can be calculated based on the Equation (2):

$$N_{bulk} = c\times V\times N_A\times\frac{\pi r^2}{S} \quad (2)$$

where N_A is the Avogadro constant, r is the radius of the laser spot, c is the concentration of R6G, and V is the volume of R6G solution dropping on the surface of the silicon surface. These parameters give N_{bulk} = 8.2525× 10^{10}. The same method is used to calculate N_{SERS}. In short, 2.5 μL of 0.1 mM R6G ethanol solution is dropped on the SERS substrate and dried in the air. Based on the Equation (2), N_{SERS} = 3.36 × 10^7.

The SERS enhancement factors of Mo_2C/MoO_x heterostructure are calculated by integrating the corresponding peak intensity, the results shows that I_{SERS} is 401828 and I_{bulk} is 1174.90. Based on Equation (1), the corresponding enhancement factors are 8.4×10^5.

In the meanwhile, the I_{SERS} and N_{SERS} correspond to different concentrations are integrated and shown below:

R6G:10^{-5}M; I_{SERS}=305181 N_{SERS}=3.36×10^6

R6G:10^{-6}M; I_{SERS}=210385 N_{SERS}=3.36×10^5

R6G:10^{-7}M; I_{SERS}=87849 N_{SERS}=3.36×10^4

R6G:10^{-8}M; I_{SERS}=4987 N_{SERS}=3.36×10^3

Based on Equation (1), the corresponding enhancement factors are 8.4×10^5 (10^{-4}-M R6G), 6.4×10^6 (10^{-5}-M R6G), 4.4×10^7 (10^{-6}-M R6G), 1.0×10^8 (10^{-7}-M R6G) and 1.8×10^8 (10^{-8}-M R6G), respectively.
